# ROC Generated Thresholds for Field-Assessed Aerobic Fitness Related to Body Size and Cardiometabolic Risk in Schoolchildren

**DOI:** 10.1371/journal.pone.0045755

**Published:** 2012-09-21

**Authors:** Lynne M. Boddy, Non E. Thomas, Stuart J. Fairclough, Keith Tolfrey, Sinead Brophy, Anwen Rees, Gareth Knox, Julien S. Baker, Gareth Stratton

**Affiliations:** 1 The Research Institute for Sport and Exercise Sciences, Liverpool John Moores University, Liverpool, United Kingdom; 2 Centre for Children and Young People’s Well-Being, College of Human and Health Sciences, Swansea University, Swansea, United Kingdom; 3 School of Sport, Exercise and Health Sciences, Loughborough University, Leicestershire, United Kingdom; 4 Cardiff School of Sport, Cardiff Metropolitan University, Cardiff, United Kingdom; 5 Department of Business and Applied Sport Science, University of the West of England (Hartpury College), Gloucester, United Kingdom; 6 Sport, Health and Exercise, School of Science, University of the West of Scotland, Hamilton, United Kingdom; 7 College of Engineering, Swansea University, Swansea, United Kingdom; 8 School of Sports Science, Exercise and Health, The University of Western Australia, Perth, Australia; Pennington Biomedical Research Center, United States of America

## Abstract

**Objectives:**

1. to investigate whether 20 m multi-stage shuttle run performance (20mSRT), an indirect measure of aerobic fitness, could discriminate between healthy and overweight status in 9–10.9 yr old schoolchildren using Receiver Operating Characteristic (ROC) analysis; 2. Investigate if cardiometabolic risk differed by aerobic fitness group by applying the ROC cut point to a second, cross-sectional cohort.

**Design:**

Analysis of cross-sectional data.

**Participants:**

16,619 9–10.9 year old participants from SportsLinx project and 300 11–13.9 year old participants from the Welsh Schools Health and Fitness Study.

**Outcome Measures:**

SportsLinx; 20mSRT, body mass index (BMI), waist circumference, subscapular and superilliac skinfold thicknesses. Welsh Schools Health and Fitness Study; 20mSRT performance, waist circumference, and clustered cardiometabolic risk.

**Analyses:**

Three ROC curve analyses were completed, each using 20mSRT performance with ROC curve 1 related to BMI, curve 2 was related to waist circumference and 3 was related to skinfolds (estimated % body fat). These were repeated for both girls and boys. The mean of the three aerobic fitness thresholds was retained for analysis. The thresholds were subsequently applied to clustered cardiometabolic risk data from the Welsh Schools study to assess whether risk differed by aerobic fitness group.

**Results:**

The diagnostic accuracy of the ROC generated thresholds was higher than would be expected by chance (all models AUC >0.7). The mean thresholds were 33 and 25 shuttles for boys and girls respectively. Participants classified as ‘fit’ had significantly lower cardiometabolic risk scores in comparison to those classed as unfit (p<0.001).

**Conclusion:**

The use of the ROC generated cut points by health professionals, teachers and coaches may provide the opportunity to apply population level ‘risk identification and stratification’ processes and plan for “at-risk” children to be referred onto intervention services.

## Introduction

The combination of excessive adiposity and poor aerobic fitness confers significant disease risk to youth [1]. The 2009 UK Chief Medical Officer’s report highlighted the importance of aerobic fitness as a health marker [2], and a growing body of literature describes aerobic fitness as a key, independent determinant of health [3]. Poor aerobic fitness and excessive adiposity are associated with similar health complications, in particular cardiometabolic disease [4]. Evidence suggests that fitness is associated with total and abdominal obesity, as well as cardiometabolic risk [3], [5]. Recent evidence has highlighted that the prevalence of childhood obesity has stabilized [6], whilst levels of aerobic or cardiorespiratory fitness have declined independent of changes in body mass index [7].

Thresholds for directly assessed aerobic fitness related to cardiometabolic risk were recently published using data from the European Youth Heart Study (EYHS) [8]. The generated cut points (37.0 mL/kg/min and 42.1 mL/kg/min for 9–10 yr old girls and boys respectively) are extremely valuable for researchers that conduct direct assessments of aerobic fitness with children. A further study using EYHS data assessed the diagnostic accuracy of a Receiver Operating Characteristic (ROC) generated cut point for aerobic fitness using direct, cycle ergometer assessments of aerobic fitness, and found the ROC cut point to distinguish effectively between those at increased risk of cardiometabolic disease [9]. The authors suggested that aerobic fitness represented an accurate method of identifying children at increased cardiometabolic risk. Another recent study developed aerobic fitness standards for detecting risk of the metabolic syndrome using data from the National Health and Nutrition Examination Survey [5]. The study used treadmill exercise tests to estimate VO_2peak_ which was then related to metabolic syndrome in 12–18 yr old participants. The resultant ROC generated thresholds for low risk ranged from 40–44 mL/kg/min for boys and 38–40 mL/kg/min for girls [5].

**Table 1:** Descriptive characteristics for the SportsLinx Cohort.

	Boys		Girls	
Measure	Mean	SD	Mean	SD
Age (years)	9.8	0.4	9.8	0.4
BMI (kg/m2)	18.2	3.3	18.5	3.5
Waist Circumference (cm)	62.3	9.8	62.1	10.2
Triceps Skinfold (mm)	14.1	6.0	16.8	5.9
Subscapular Skinfold (mm)	8.9	6.1	11.1	6.9
20mSRT performance (shuttles)	39.8	19.4	27.6	13.5

Despite evidence from these studies, the majority of aerobic fitness assessments in children are undertaken on a large-scale, and are completed in the field often using 20 m multi-stage shuttle runs tests (20mSRT). This approach is pragmatic at a population level and overcomes the technical, time and cost demands of directly assessed aerobic fitness. Field-based, indirect assessments of aerobic fitness are widely used within screening programmes at the school level. In response to calls from the UK Chief Medical Officer for more fitness testing in children [2] it is likely that field-tests of aerobic fitness will become more commonplace. The ability to discriminate between a ‘healthy’ and ‘unhealthy’ level of aerobic fitness related to body size by simply comparing 20mSRT performance to a cut point would be of significant public health value. Cut points for 20mSRT data would be especially useful if they can provide health professionals with a given value that represents increased risk, not only of excessive adiposity, but also for clustered cardiometabolic risk. Furthermore, by evaluating aerobic fitness using thresholds, risk ‘stratification’ may be completed, and those identified as potentially at risk can be referred on to the relevant intervention or programme [9].

**Table 2:** ROC curve analyses for boys.

Model	N	AUC	95% CI	Cut point	Estimated VO_2peak_	Sensitivity	1-Specificity
1 (BMI)	6810	0.782	.770–.794	32.5 shuttles	46.6 mL/kg/min	.703	.268
2 (Waist)	3771	0.736	.720–.753	34.5 shuttles	46.6 mL/kg/min	.683	.325
3 (%BF)	6252	0.800	.787–.813	32.5 shuttles	46.6 mL/kg/min	.667	.195

**Table 3:** ROC curve analyses for girls.

Model	N	AUC	95% CI	Cut point	Estimated VO_2peak_	Sensitivity	1-Specificity
1 (BMI)	6676	.752	.740–.764	24.5 shuttles	41.9 mL/kg/min	.626	.248
2 (Waist)	3843	.720	.704–.736	26.5 shuttles	41.9 mL/kg/min	.578	.285
3 (%BF)	6149	.772	.758–.787	23.5 shuttles	41.9 mL/kg/min	.612	.182

To date, no studies have investigated whether a given level of field-based, indirectly assessed aerobic fitness is associated with healthy body weight, and/or cardiometabolic risk. Therefore the aims of this study were to:

1. Investigate whether 20mSRT performance could discriminate between healthy and overweight status in 9–10.9 yr old schoolchildren using ROC analysis.
2. Investigate if cardiometabolic risk differed by 20mSRT threshold group by applying the ROC generated cut point to a second, cross-sectional cohort.

## Materials and Methods

Data were generated for the ROC analysis from the Liverpool SportsLinx database, the methods have been described elsewhere [7], [10]. Briefly, all schools in the Liverpool Local Education Authority were invited to take part in a SportsLinx fitness fun day during the study period. For each participating school, all Year 5 school children (9–10.9 yrs of age) were invited to take part. Typically, SportsLinx recruits ∼75–85% of all Y5 children, and is representative of the local population. Children attended a fitness fun day with their school class at a local school hall, where they completed a fitness testing battery adapted from Eurofit [11]. SportsLinx anthropometric and 20mSRT measures have been collected year on year by two trained and experienced Fitness Officers since 1998 and have acceptable test/re-test reliability [12]. For the ROC analysis component of this study, data were included for 16,619 (N = 8382 boys) participants from five consecutive school years 2005/6 to 2009/10. Variables included in analysis were: stature, body mass, waist circumference, %fat calculated from triceps and subscapular skinfold thicknesses using Slaughter equations [13], and performance on the 20mSRT. Three ROC curve analyses were completed, separately by sex. Each assessed whether 20mSRT performance (number of completed shuttles) could discriminate between healthy and overweight status. To complete analyses, participants were classified as normal weight or overweight using: the international definition of overweight using BMI [14], the 90^th^ percentile for waist circumference [15] and by calculating percentage body fat (%BF) for skinfolds using pre-pubescent equations [13], where 25% and 30% represented overweight for boys and girls, respectively. These %BF thresholds were selected as they have been related to cardiometabolic risk in children and adolescents in previous research [16]. ROC model one included 20mSRT and BMI status, model two included 20mSRT and waist circumference status, and model three included 20mSRT and %BF status. The number of completed shuttles were identified as thresholds and mean decimal age for the cohort were used to estimate VO_2peak_ using regression equations [17]. The second set of cross-sectional data was taken from a field-based study conducted in South Wales, UK, the Welsh Schools Health and Fitness Study. The protocols for this study have been described elsewhere [18]. Data were collected by two trained researchers, and variables included in the present study included: sex, age, waist circumference, 20mSRT performance, blood pressure and fasting venous blood sample markers. Clustered risk scores were calculated for boys and girls separately by standardising risk variables and summing them to create a risk score, the components of the risk score are presented below. Studies have often created a clustered risk score for cardiometabolic risk, as differences in individual risk markers between participants may be too subtle to investigate in isolation, and a clustered score may compensate for daily fluctuations in markers [8]. Furthermore, the metabolic syndrome and cardiovascular disease are characterised by a constellation of risk markers, and, therefore, a clustered risk score may detect the array of cardiometabolic disturbances rather than focussing on one or two particular markers.

**Figure 1:**
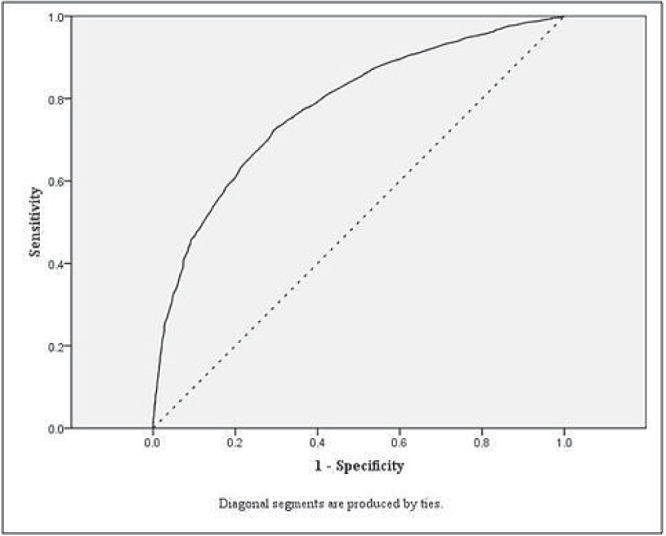
ROC curve for model one, Boys (BMI and 20mSRT).

**Figure 2:**
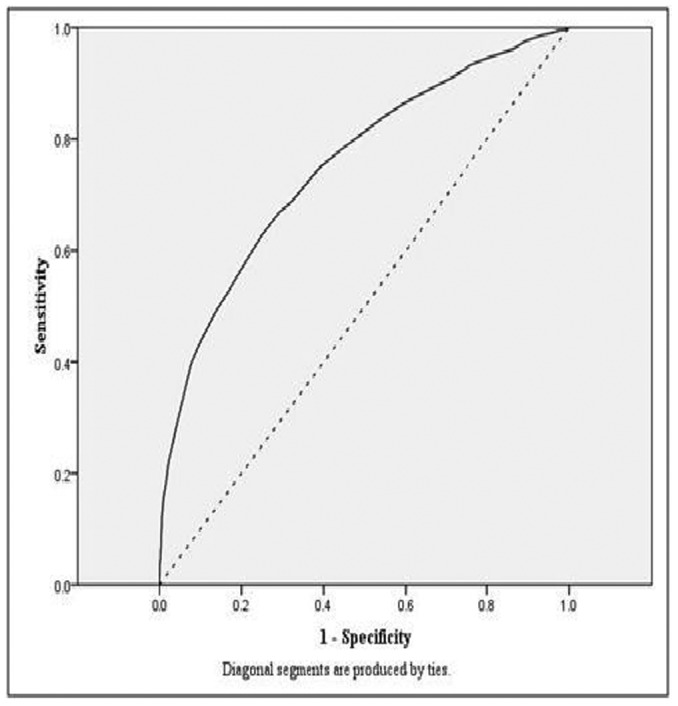
ROC curve for model one, Girls (BMI and 20mSRT).

The following variables violated the assumptions of normality and were log-transformed prior to standardisation, then back transformed for presentation purposes: girls; waist circumference, systolic blood pressure (BP), diastolic BP, total cholesterol to high density lipoprotein cholesterol (HDL-C) ratio (TC:HDL-C), glucose, insulin, adiponectin, C-reactive protein (CRP). Boys; waist circumference, systolic BP, diastolic BP, TC:HDL-C, glucose, insulin, CRP, adiponectin.

**Table 4:** Descriptive characteristics for the Welsh Schools Health and Fitness Study Cohort.

	Boys	Girls
Age (years)	12.6 (0.8)	12.6 (0.7)
Stature (m)	1.6 (0.1)	1.5 (0.1)
Body Mass (kg)	49.0 (13.0)	50.0 (11.6)
BMI (kg/m^2^)	20.1 (3.8)	20.9 (3.9)
Waist Circumference (cm)	68.9 (9.8)	68.7 (10.0)
20 m SRT Score (shuttles)	53.5 (22.2)	37.2 (15.9)
Systolic Blood Pressure (mmHg)	115.6 (12.7)	114.6 (12.8)
Diastolic Blood Pressure (mmHg)	66.2 (11.5)	66.8 (11.1)
Total Cholesterol : HDL	2.7 (0.7)	2.8 (0.9)
Glucose (mmol/L)	4.9 (0.4)	4.9 (0.4)
Insulin (mmol/L)	8.8 (8.1)	11.0 (7.4)
Adiponectin	3354.2 (2040.5)	4275.1 (2495.3)
C-Reactive Protein	1.1 (2.8)	0.9 (1.7)

Two scores were calculated, including the following variables: Score 1. Waist circumference, systolic and diastolic BP, TC:HDL-C, insulin, glucose, adiponectin (inverted), CRP. Score 2. Waist circumference, systolic and diastolic BP, TC:HDL-C, insulin, glucose. The second score omitted CRP and adiponectin to maximise participant numbers. Selection of risk variables was based on the components of the International Diabetes Federation definition for metabolic syndrome [19], and CRP and adiponectin were included as both are potent markers of CVD risk [18].

**Table 5:** Mean risk scores (SE) by fitness status and sex.

	Fit			Unfit		
	Group	Boys	Girls	Group	Boys	Girls
Clustered Risk Score 1	−1.13 (.26)*N = 220	−1.53 (.39)*N = 90	−.85 (.34)*N = 130	3.04 (.44)N = 77	3.98 (.70)N = 28	2.50 (.55)N = 49
Clustered Risk Score 2	−.76 (.19)*N = 300	−.98 (.30)*N = 125	−.60 (.25)*N = 175	1.97 (.34)N = 98	2.41 (.56)N = 36	1.72 (.42)N = 62

* Fit < unfit, p≤0.001.

### Ethics Statement

The SportsLinx study (NRES Committee North West, Liverpool East) and the Welsh Schools Health and Fitness Study (Dyfed Powys Research Ethics Committee) have full ethical approvals from the respective Local NHS Research Ethics Committees. Informed, written parental consent and participant assent was obtained prior to participation for all children involved in the studies.

### Statistical Analysis

Area under the curve (AUC) statistics and 95% confidence intervals were recorded for the ROC curve analyses. The CRF threshold or cut point for each model was defined as the co-ordinate that had the closest value to 1 for the difference between the true positive (sensitivity) and false-positive (1-specificity) values. The mean of the three generated CRF thresholds/cut points (number of completed shuttles) was calculated by sex and retained for further analysis. Participants from the Welsh Schools Health and Fitness Study were then classed as fit or unfit using the generated sex-specific cut points and analysis of covariance (age and sex as covariates) was completed to assess any differences in risk by fitness status for the whole group, and separately by sex (age as covariate). All analyses were conducted using SPSS V. 17 (SPSS Inc. Chicago, IL), and an alpha value of p≤0.05 was used to denote statistical significance.

## Results

### 1. ROC Curve Analyses

The descriptive characteristics for the SportsLinx participants are displayed in Table 1. According to age and sex specific BMI cut points, 30.6% and 25.2% of girls and boys were classified as overweight. In terms of ethnicity, 85.8% of the cumulative SportsLinx sample were classified as White British.

The AUC, 95% confidence intervals and identified cut points (co-ordinate closest to 1), plus sensitivity and 1-specificity values are displayed in Tables 2 and 3 for the three ROC curve analyses by sex.

Figures 1 and 2 display the model one ROC curves for boys and girls, respectively. The diagnostic accuracy of the cut points for identifying children at risk of overweight by all measures was higher than what would be expected by chance (for all models AUC >0.7).

The highest AUC values were observed for the per cent body fat models. Identified cut points were similar across the three models for boys and girls, and the mean cut point was 33 shuttles (mean of the three cut points  = 33.2 shuttles) for boys and 25 shuttles for girls (mean of the three cut points  = 24.8 shuttles).

### 2. Applying CRF Thresholds to Cardiometabolic Risk Data

Table 4 displays the descriptive characteristics of the Welsh cohort. For the whole cohort the prevalence of overweight was 31.2% for girls and 26.7% for boys. For the Welsh cohort, 87.2% of participants were classified as White British.

Clustered risk scores by fitness status and sex are displayed in Table 5. For both clustered risk scores, at the group level and separately by sex, those classified as fit had lower risk scores in comparison to those classed as unfit using the ROC generated cut points (p<0.001).

## Discussion

The aims of this study were to: 1. investigate whether 20mSRT performance could discriminate between healthy and overweight status in 9–10.9 yr old schoolchildren using ROC analysis, 2. investigate if cardiometabolic risk differed by the aerobic fitness threshold group by applying the ROC generated cut point to a second, cross-sectional cohort.

The generated ROC curves displayed acceptable AUC and 95% confidence interval limits, suggesting the resultant thresholds were not due to chance (all AUC >0.7) and effectively distinguished between overweight and normal weight participants on the basis of 20mSRT performance. The thresholds generated using three estimates of body size/fatness were similar, and the mean value from the analyses provides a simple aerobic threshold in boys and girls for practitioners to use in the field. The values proposed in the present study (<33 shuttles for boys [estimated VO_2peak_; 46.6 mL/kg/min] and <25 shuttles [estimated VO_2peak_; 41.9 mL/kg/min] for girls) are higher than those suggested by Ruiz et al [8] (42.1 mL/kg/min for boys and 37.0 mL/kg/min for girls) and Adegoboye et al (43.6 mL/kg/min for boys and 37.4 mL/kg/min for girls) [9]. In the present study VO_2peak_ was estimated indirectly through 20mSRT performance rather than by direct assessment, and these differences in VO_2_ thresholds may reflect the alternative data collection processes (e.g. online gas analysis systems, cycle ergometer vs running assessments) utilised, and the body size measures used to determine the thresholds.

When applied to the Welsh Schools Health and Fitness Study cohort, participants classified as ‘low aerobically fit’, displayed significantly higher clustered cardiometabolic risk scores, than those who reached the aerobic fitness threshold. Other studies have described increased cardiometabolic risk in less aerobically fit individuals [20], [21]; however, these results are not from field-based assessments of aerobic fitness. The findings presented in this study describe thresholds that are supported by clinical markers, which when applied to a separate, differently aged cohort from the population that the ROC curves were originally generated from, showed differences in cardiometabolic risk between those reaching the thresholds and those who did not. The thresholds and the association between these ‘cut points’ and cardiometabolic risk provide a clear insight into the links between aerobic fitness and disease risk, and highlight the need for public health interventions to promote aerobic fitness. Such interventions may involve promoting vigorous physical activity to stimulate aerobic fitness [3], [7], which has been alluded to in the most recent UK CMO guidelines for physical activity [22]. These thresholds are the first to be based on the widely used 20mSRT assessment, and provide a highly useful method of classifying aerobic fitness/risk in children.

There are some limitations to this study. Despite the large SportsLinx sample size, the age range included within analyses is narrow (9–10 years old), and therefore the application of the thresholds to a wider age range requires further investigation. However, when thresholds were applied to the Welsh Schools Health and Fitness Study cohort, who were slightly older children (11–14 years old), significant differences in clustered cardiometabolic risk scores were apparent, suggesting that there is value in applying the thresholds in the absence of cut points calculated specifically for a slightly older age-group. Similar studies are required to examine cut points across the childhood age range, and merging datasets from numerous field-based fitness studies that have utilised standard methods may be required to achieve this. A further limitation is the lack of control for maturation status. The ROC analysis approach does not allow confounding to be accounted for within the model, and data were not adjusted prior to ROC analysis. Future studies should aim to include maturation within analyses. Finally, the extent to which the association between aerobic fitness and weight represents collinearity is unknown and warrants further investigation in future studies.

The major strength of this study is the potential utility of the thresholds for use in field settings by practitioners. As the 20mSRT is a simple, low cost method of assessing aerobic fitness its use is urged at a population level, whilst using the aerobic fitness thresholds to allow risk stratification and effective identification of individuals in need of physical activity and fitness promotion.

The use of the ROC generated cut points in practice by General Practitioners and other health professionals, teachers and coaches may ensure that children can be referred onto intervention services effectively using a simple, low cost aerobic fitness assessment in the field.
